# Basal cell carcinoma, squamous cell carcinoma and melanoma of the head and face

**DOI:** 10.1186/s13005-016-0106-0

**Published:** 2016-02-05

**Authors:** L. Feller, R. A. G. Khammissa, B. Kramer, M. Altini, J. Lemmer

**Affiliations:** Department of Periodontology and Oral medicine, Sefako Makgatho Health Sciences University, Medunsa, 0204 South Africa; School of Anatomical Sciences, Faculty of Health Sciences, University of the Witwatersrand, Johannesburg, South Africa; Division of Anatomical Pathology, School of Pathology, University of the Witwatersrand, Johannesburg, South Africa; Department Periodontology and Oral Medicine, Box D26 School of Dentistry, Sefako Makgatho Health Sciences University, Medunsa, 0204 South Africa

**Keywords:** Basal cell carcinoma, Keratinocytes, MC1R, Melanocytes, Melanoma, Squamous cell carcinoma, Ultraviolet light

## Abstract

Ultraviolet light (UV) is an important risk factor for cutaneous basal cell carcinoma, cutaneous squamous cell carcinoma and cutaneous melanoma of the skin. These cancers most commonly affect persons with fair skin and blue eyes who sunburn rather than suntan. However, each of these cancers appears to be associated with a different pattern of UV exposure and to be mediated by different intracellular molecular pathways.

Some melanocortin 1 receptor (MC1R) gene variants play a direct role in the pathogenesis of cutaneous basal cell carcinoma, cutaneous squamous cell carcinoma and cutaneous melanoma apart from their role in determining a cancer-prone pigmentory phenotype (fair skin, red hair, blue eyes) through their interactions with other genes regulating immuno-inflammatory responses, DNA repair or apoptosis.

In this short review we focus on the aetiological role of UV in cutaneous basal cell carcinoma, cutaneous squamous cell carcinoma and cutaneous melanoma of the skin, and on some associated biopathological events.

## Background

The skin of the head and face is habitually exposed to sunlight and there may consequently be ultraviolet (UV)-induced chronic damage such as solar elastosis, actinic keratosis and lentigo, with possible consequent development of cutaneous squamous cell carcinoma, cutaneous basal cell carcinoma and cutaneous melanoma [1].

Cutaneous squamous cell carcinoma originates from stem/progenitor cells of the basal cell layer of the epidermis and cutaneous basal cell carcinoma from either the bulge region of hair follicle which is rich in keratinocyte stem cells or from stem/progenitor cells of the basal cell layer of the epidermis [2, 3]. While the origin of melanoma cells is unknown, it has been proposed that cutaneous melanoma precursor cells may arise either from dedifferentiated melanocytes or from melanocyte progenitors in the bulge region of hair follicles, or from neural crest-derived Schwann cell precursors [1–4].

Cutaneous squamous cell carcinoma of the skin is associated with frequent moderate chronic UV exposure, and is often preceded by premalignant actinic keratosis or by Bowen disease. On the other hand, cutaneous basal cell carcinoma is usually associated with intermittent, infrequent, intense UV exposure and almost invariably arises *de novo* [1, 5–7]. Cutaneous melanoma can also arise *de novo* after intermittent, infrequent, intense UV exposure, but there is evidence that as many as 30 % of cases evolve from pre-existing sites of melanotic pigmentation, whether or not they have actually been exposed to UV [4].

Both cutaneous squamous cell carcinoma and melanoma are very likely to metastasize whereas cutaneous basal cell carcinoma very seldom does [5]. Even in the absence of exposure to UV, squamous cell carcinoma is occasionally associated with non-healing wounds or scarring, or with chronic lesions which are, or have been preceded by chronic immuno-inflammatory processes. This is not the case with cutaneous basal cell carcinoma or cutaneous melanoma. Cutaneous squamous cell carcinoma, basal cell carcinoma and melanoma more frequently affect elderly, red haired, blue eyed and fair complexioned persons [7], and it has been consistently demonstrated that variants of the highly polymorphic melanocortin 1 receptor (MC1R) gene are associated with increased risk of these malignancies [8].

The pathogenesis of cutaneous squamous cell carcinoma is associated with multiple local genetic alterations that may bring about dysregulation of the cell cycle, of apoptosis, of DNA repair, of cellular differentiation, of telomerase activity with evasion of cellular senescence, and of expression of the enzyme cyclo-oxygenase 2 (COX-2).

In contrast, cutaneous basal cell carcinoma is primarily driven by genetic mutations causing uncontrolled activation of the hedgehog intracellular pathway leading to enhanced proliferative capacity of basal cells, and by molecular alterations in the p53 tumour-suppressor gene [9]. Most cases of cutaneous basal cell carcinoma occur sporadically, but they may also occur as a manifestation of the rare heritable basal cell nevus syndrome in association with germline molecular aberrations of the hedgehog intracellular signalling pathway [5].

Melanomas of the skin of the head and face are usually of the lentigo maligna type which is commonly associated with frequent, moderate chronic UV exposure. This is in contrast to melanomas of the trunk, for example, which are associated with intermittent, acute UV exposure. Skin melanoma cells show molecular alterations of the RAS-BRAF-MEK-ERK mitogen activated protein kinase (MAPK) signalling pathway, mediating uncontrolled proliferation of the affected malignant melanocytes; genetic alterations in the CDKN2A gene encoding the p16INK4A tumour suppressor protein; and MC1R genetic polymorphism [1, 10, 11]. In 10-30 % of cases, cutaneous melanoma arises from pre-existing melanotic hyperpigmentations such as lentigo, freckles or pigmented naevi. Despite an established cause-and-effect relationship between the development of melanoma and exposure to UV, genes with UV-induced ‘signature mutations’ are not common in melanoma [1]. Thus, it is evident that the aetiopathogenesis of melanoma is complex, with UV playing a critical role, but UV by itself does not necessarily cause melanoma. Indeed, in Black people, melanomas mainly affect body sites that are not exposed to UV such as the soles of the feet and the palms of the hands (acral melanoma) [12].

The purpose of this short review is to shed some light on the relationship between exposure to UV and cutaneous squamous cell carcinoma, basal cell carcinoma, and melanoma, and to discuss some aspects of the biopathology of these cancers.

## Melanocortin 1 receptor (MC1R) and its role in the pathogenesis of cutaneous squamous cell carcinoma, cutaneous basal cell carcinoma and cutaneous melanoma

The MC1R gene plays an important role in melanin production and in skin pigmentation. Pro-opiomelanocortin (POMC) and its derivatives, particularly α-melanocyte stimulating hormone (αMSH) are agonistic ligands of MC1R on melanocytes, mediating the biosynthesis of both red-yellow pheomelanin and brown-black eumelanin. Melanins are synthesized in melanosomes which are transported to the extremities of the melanocytic dendrites *via* microtubuli, and are ultimately transferred to neighbouring keratinocytes where they protect keratinocytes from UV-induced DNA damage [13, 14].

The MC1R gene is highly polymorphic among White people with some genetic variants mediating the production of more pheomelanin and less eumelanin, resulting in the phenotype of red hair, blue eyes and fair skin [13, 15]. Persons with a phenotype mediated by one of these MC1R genetic variants are at greater risk of UV-induced skin cancers, because pheomelanin not only provides less effective protection against UV than does eumelanin, but it also generates more mutagenic free radicals in response to UV.[16] Apart from their role in determining a cancer-prone pigmentory phenotype, it has been demonstrated that certain MC1R variants play a direct role in the pathogenesis of cutaneous squamous cell carcinoma, cutaneous basal cell carcinoma and cutaneous melanoma [5, 13, 16]. In fact, about 15 % of all cases of cutaneous melanoma are associated with some MC1R variants [17].

Non-pigmentory functions of MC1R mediated *via* the αMSH/MC1R pathway include regulation of local immuno-inflammatory responses brought about by several factors including modulation of NF-κB which is an important regulator of the production of inflammatory mediators [18, 19], mediation of the proliferation and survival of melanocytes [20], induction of DNA repair following UV-induced DNA damage [19–21], and diminution of oxidative stress by reducing the generation of reactive oxidative species that have the capacity to cause oxidative damage to cellular DNA [22].

In response to DNA damage caused by UV, there is stimulation of the keratinocytic p53 gene leading to transcriptional activation of αMSH inducing DNA repair mechanisms mediated through xeroderma pigmentosum proteins [23, 24]. In melanocytes, UV induces both the upregulation of expression of MC1R and the production of αMSH, that *via* the MC1R/αMSH/cAMP pathway activates DNA repair mechanisms [21] and diminishes oxidative stresses [22, 25]. This prevents the development of genomic instability in response to UV, and minimizes the risk of UV-induced malignant transformation of melanocytes [22].

These non-pigmentory functions outlined in the above two paragraphs are dysregulated in melanocytes expressing those MC1R variants, thus promoting the risk of melanoma [13, 16, 20, 21]. In fact, compared to melanocytes with mainstream MC1R it has become clear that in response to UV-induced DNA damage, melanocytes with MC1R variants have a lower DNA repair capacity, more DNA mutagenic photoproducts, increased oxidative DNA damage, and decreased apoptosis [21, 25]. Thus, the risk of melanoma is polygenetic comprising interactions between MC1R variants, other pigmentory gene variants, dysfunctional DNA repair genes and immuno-inflammatory genes [19, 26].

## Cutaneous squamous cell carcinoma

Cutaneous squamous cell carcinoma frequently affects elderly White people with a phenotype of red hair, blue eyes and fair skin, who for a long time have been chronically exposed to UV. In keratinocytes, UV induces two major classes of mutagenic photoproducts: cyclobutane-pyrimidine dimers (CPDs), and 6,4 pyrimidine-pyrimidine. These DNA lesions may give rise to the genetic mutations C → T and/or CC → TT that are the hallmarks of UV-induced mutagenesis, and are considered to be ‘UV signature mutations’ [12, 27, 28]. Furthermore, in sun-exposed skin, UV can induce the generation of highly reactive oxygen species with the capacity to cause DNA damage, thus further promoting mutagenesis [27, 29].

In general, if the damage to DNA affects oncogenes, tumour-suppressor genes (anti-oncogenes), or cell cycle checkpoint control genes, cellular genomic integrity will be destabilized with increased risk of acquiring additional cytogenetic alterations. Molecular alterations in oncogenes may permit uncontrolled cell proliferation in response to microenvironmental growth signals; and in tumour suppressor genes may result in dysregulated oncogenic activity. Molecular alterations in cell cycle checkpoints may prevent arrest of the cell cycle that is necessary to allow DNA repair, or may prevent apoptosis in response to DNA damage which exceeds the cellular DNA repair capacity, with the outcome of propagation of the altered DNA by cell division of the transformed cells [30, 31]. Since some of these transformed cells are metabolically fitter and have a selective growth advantage over the normal neighbouring cells [32], they will ultimately undergo clonal expansion, and spread at the expense of the normal surrounding keratinocytes [31], creating a field or multiple fields of precancerized epidermis comprising cytogenetically-altered keratinocytes at different stages of transformation.

Such a field of precancerized epidermis with molecularly altered keratinocytes may look clinically normal and may or may not have the microscopical features of UV-induced damage (i.e. solar elastosis, epidermal dysplasia), or may exhibit the clinical and histopathological features of actinic keratosis which is a precursor lesion of cutaneous squamous cell carcinoma [33]. Subsequent clonal divergence will result in the evolution of subclones which would have had multiple episodes of genetic mutations, one or more of which will eventually give rise to frank cutaneous squamous cell carcinoma in all its clinical variety [33].

The dermis/stroma plays an important role in the pathogenesis of squamous cell carcinoma. At the interface between epidermis and dermis/stroma, lies the basement membrane which initially prevents the tumour from invading the underlying connective tissue. The degradation of collagen, particularly collagen type IV, in the basement membrane is crucial for the infiltration of the underlying connective tissue by the tumour cells and is achieved by the matrix metalloproteinases (MMP)-2 and 9 [34]. Increased expression of MMP-2 is found in the stroma of squamous cell carcinoma compared with that of basal cell carcinoma and may thus be responsible for the different pattern of invasion found in these two tumours [35]. Metastasis to lymph nodes is also said to be assisted by these MMPs [34, 36]. Depletion of collagen VI, which is a component of the fibrils which anchor the basement membrane to the dermis, promotes invasion and epithelial-mesenchymal transition of keratinocytes [37].

Once the basement membrane has been breached by the tumour cells, they become associated with a multicellular tumoural environment which is essential in modulation of the tumour and in its metastasis. An important cell type in this milieu is the cancer-associated (myo-) fibroblast (CAF), identified by the presence of α-smooth muscle actin [38, 39] and which occurs profusely in the tumour environment [40]. The restructuring of the extracellular matrix which facilitates invasion occurs by the production of chemokine ligand 7 by the CAFs [41]. This is in contrast to connective tissue growth factor (CTGF) which promotes mesenchymal to epithelial transition and suppresses invasiveness [42] in head and neck squamous cell carcinoma.

## Cutaneous basal cell carcinoma

Cutaneous basal cell carcinoma is the most common cancer in White people [9, 43]. It is so named because the cells of basal cell carcinoma resemble the cells of the basal cell layer of the epithelium [5]. Cutaneous basal cell carcinoma affects mainly chronically sun-exposed skin of the head and neck of fair complexioned older people. Both intermittent acute, and long standing continual exposure to UV are high risk factors for cutaneous basal cell carcinoma. It is a slow-growing cancer that if left untreated will invade locally, but only rarely metastasizes. Black people are rarely affected. People with cutaneous basal cell carcinoma are at increased risk for cutaneous squamous cell carcinoma and cutaneous melanoma [5, 9, 11, 43].

Dysregulation in the hedgehog intracellular signalling pathway is implicated in the pathogenesis of cutaneous basal cell carcinoma and is thought to be an early genetic factor in its tumourigenesis [44]. The hedgehog signalling pathway plays an essential role in organogenesis, and later, postnatally, in regulating proliferation and differentiation of keratinocyte stem cells, and in the development of hair follicles and sebaceous glands [1]. In the hedgehog signalling pathway, patch 1 (PTCH1) functions as a tumour suppressor gene, inhibiting the activity of the proto-oncogene, smoothened (SMO). Signalling by SMO results in activation of transcription of hedgehog target genes, eliciting mitogenic responses with increased proliferation of keratinocyte stem cells. Clonal expansion of dysregulated cells within the keratinocytes stem cell niche is favoured by loss-of-function mutation in PTCH1 that allows upregulated activity of SMO, and by gain of function mutations in the SMO gene that render SMO protein resistant to inhibition by PTCH1. These promote escape of cancer precursor cells from the niche to colonize other cellular compartments in the skin, ultimately giving rise to a basal cell carcinoma [9, 11, 45].

The molecular alterations in the hedgehog signalling pathway may be of germline origin or may occur postnatally subsequent to UV-induced DNA damage, or rarely may arise spontaneously [11, 46]. Keratinocytes which show dysregulated expression of the hedgehog signalling pathway fail to undergo cell-cycle arrest in response to the p21 cell cycle inhibitor, and thus have enhanced proliferative capacity [6]. It has been reported that most cases of cutaneous basal cell carcinomas that arise spontaneously show loss of function of PCTH1 and a minority show enhanced function of SMO [5]. About half of all cases of sporadic basal cell carcinoma also show mutations in the p53 tumour suppressor gene, but these seem to be late genetic events in the tumourigenesis of cutaneous basal cell carcinoma, which are related to its progression [45, 47]. Taking advantage of the inhibition of the hedgehog signalling pathway as a mean of treatment of cutaneous basal cell carcinoma of the skin has become possible through the use of an inhibitor, vismodegib (GDC-0049) and other hedgehog pathway antagonists [48].

Cutaneous basal cell carcinoma requires a specific stromal environment to maintain its morphological characteristics [49]. Key regulators of the biological behaviour of cutaneous basal cell carcinoma appear to be stromal fibroblasts and myofibroblasts [49]. Cutaneous basal cell carcinoma cells express bone morphogenetic protein (BMP) 2 and 4, while GREMLIN 1, a BMP antagonist is highly expressed in the stroma of the tumour but not in the dermis underlying normal keratinocytes. GREMLIN 1 counteracts the growth-inhibitory effect of BMPs and is therefore assumed to be an important agent supporting cutaneous basal cell carcinoma cell proliferation and survival. Matrix metalloproteinase expressed in the stroma of cutaneous basal cell carcinoma also plays an important role in regulating growth and other functions of cutaneous basal cell carcinoma cells [6]. It is probable that cutaneous basal cell carcinoma tumourigenesis depends substantially on specific factors produced by stroma damaged by UV, but on the rare occasions when cutaneous basal cell carcinomas occur at sites that are not exposed to sunlight, other biological factors drive its initiation and progression [5].

Although UV-radiation is a primary aetiological factor for cutaneous basal cell carcinoma, the mechanism whereby exposure to UV triggers cutaneous basal cell carcinoma is complex and the details are as yet unknown [5]. The development of cutaneous basal cell carcinoma at sites not exposed to UV is unexplained. Cutaneous basal cell carcinoma usually develops at sites continually exposed to UV which explains why basal cell carcinoma so frequently affects the skin of the head and face [5, 9, 50, 51].

Reduced functional activity of genes regulating repair of DNA damaged by UV may be a modifying factor in the pathogenesis of cutaneous basal cell carcinoma [5]. In support of this, subjects with the genetic condition, xeroderma pigmentosum in whom there are high-penetrance germline mutations in genes encoding proteins involved in the mechanism of nucleotide excision-repair are at great risk of developing multiple and recurrent cutaneous basal cell carcinomas at a young age [5, 52].

In contrast to subjects not affected by basal cell carcinoma, those who do have basal cell carcinoma show reduced clearance of mutagenic photoproducts from UV-induced DNA lesions of sunlight exposed skin [5], probably owing to low-penetrance genetic polymorphisms of specific genes encoding enzymes involved in breaking down reactive oxygen species and DNA repair [32].

## Cutaneous melanoma

Cutaneous melanoma is an aggressive skin tumour that can be classified into 4 main subtypes: superficial spreading, nodular, lentigo maligna and acral melanoma. Lentigo maligna melanoma typically affects long-term chronically UV-exposed skin of the head and face [53], where other subtypes are relatively rare [29]. The frequency of lentigo maligna melanoma increases with age and peaks in the seventh to eighth decades of life [10, 54]. Lentigo maligna is an epithelial field of atypical melanocytes which takes the form as an ill-defined brown macule which slowly expands centrifugally. When these atypical epithelial melanocytes breach the basement membrane and invade the connective tissue, the lesion is referred to as lentigo maligna melanoma [54]. Cutaneous melanomas of the head and neck are less likely to arise in association with pre-existing pigmented nevi than cutaneous melanomas of the trunk [55].

Many complex factors are implicated in the pathogenesis of cutaneous melanoma including family history, phenotypic characteristics such as pale skin and red hair with propensity to sunburn, many episodes of sunburn especially in youth, the presence of numerous melanotic nevi or freckles, and pre-existing dysplastic nevi. Further factors are MC1R genetic polymorphism, and perhaps other yet ill-defined environmental factors [29, 56], but some MC1R variants are associated with increased risk of cutaneous melanoma regardless of skin type and hair colour [16, 20].

Like cutaneous squamous cell carcinoma, lentigo maligna melanoma typically affects chronically UV-exposed skin of the head and face, but the risk of other subtypes of melanoma is more related to intermittent, intense UV exposures [51, 53, 54].

In contrast to malignant keratinocytes of cutaneous squamous cell carcinoma that show UV-induced signature-mutations, these are rare in cutaneous melanoma cells [12], and while mutations to tumour-suppressor gene p53 are frequent in UV-induced squamous cell carcinoma, in UV-induced cutaneous melanoma they are not [12]. Cutaneous melanoma cells but not malignant keratinocytes show oncogenic mutations in either NRAS or BRAF. Except for acral and mucosal melanomas, BRAF mutations are an early genetic event of melanoma tumourigenesis and can be found in up to 60 % of frank melanomas [22, 57]. In contrast, in mucosal and acral melanomas there are gain-of-function mutations in the cKit receptor thyrosine kinase [4]. Inactivating mutations in the CDKN2A gene which encodes for p16INK4a tumour suppressor protein, pose a high risk for development of cutaneous melanoma [12, 58]. Both BRAF and CDKN2A mutations in cutaneous melanoma cells are characteristic of indirect UV-induced oxidative damage [25].

Cutaneous melanoma arising from melanocytes residing in the basal cell layer of the epidermis usually shows one of two histopathological patterns. In one pattern there is a radial growth phase characterized by proliferation of atypical melanocytes within the epidermis and by small breaches of the basement membrane. In the other pattern there is a phase of ‘vertical’ growth which occurs once the basement membrane is breached and the melanoma starts to invade the dermis in a nodular pattern without any significant preceding phase of radial growth [4]. A further third pattern of growth occurs when the cutaneous melanoma originates from melanocyte precursors that reside in the dermis as a result of arrest in their migration from the neural crest. In this case, epidermal melanocytes do not contribute to the cutaneous melanoma and there is no evidence of invasive breaching of the basement membrane [4].

Cutaneous melanoma is rare in Black persons, and when it occurs, it preferentially affects body sites not habitually exposed to UV such as the sole of the foot, the palm of the hand and the nail bed (acral melanomas). Although the dorsum of the hand is usually constantly exposed to UV-radiation, for some obscure reasons it is only rarely affected by cutaneous melanoma [53]. Curiously, while the frequency of cutaneous basal cell carcinoma and cutaneous squamous cell carcinoma is high in albino Blacks, cutaneous melanoma is rare in this population group [59, 60].

There is evidence of loss of integrity of membranes of the melanosomes in melanoma cells with consequent leakage of reactive oxygen species (ROS) and metabolic by-products of melanogenesis that may be cytotoxic, genotoxic or mutagenic into the cytoplasm of melanoma cells contributing to progressive DNA damage [61]. It is possible that the primary alterations to DNA, favouring initial transformation of normal melanocytes and later promoting malignant transformation of the initially transformed melanocytes may result from a similar loss of integrity of the membranes of melanosomes within normal melanocytes [4, 61, 62].

## UV-induced immunosuppression

It is known that the immune system has the capacity to recognize and to react to immunogenic cells in any population of tumour cells. T cell-mediated immune responses against tumour-specific antigens may directly cause lysis of tumour cells, and activated inflammatory cells may non-specifically destroy tumour cells by producing and secreting active biological mediators [63].

Both in dark and in light skin, UV can cause local immune suppression by altering antigen presentation by Langerhans cells, and by increasing expression of immunosuppressive neuropeptides, melanocortins, cytokines and inflammatory mediators released into the microenvironment by keratinocytes, melanocytes, neurocytes and mast cells [29, 64, 65]. This may diminish the capacity of the immune system to detect and to eliminate immunogenic initially transformed keratinocytes and melanocytes, and so to promote the growth of cutaneous basal cell carcinoma, cutaneous squamous cell carcinoma and cutaneous melanoma [29].

## Conclusion

The risk of non-syndromal cutaneous basal cell carcinoma, cutaneous squamous cell carcinoma and cutaneous melanoma is associated with UV exposure, but the complex multifactorial relationship between the patterns of exposure to UV and the pathogenesis of these skin cancers is yet to be explained. However, it is established that polymorphic low-penetrance pigmentory genes governing pigmentation, genes involved in the mechanisms of breaking down UV-induced reactive oxygen species, genes encoding DNA-repair proteins, and UV-induced genetic mutations, all interact on a background of UV-induced local immunosuppression and other genetic and environmental factors in the initiation and progression of these malignancies.

Thus, susceptibility to sporadic cutaneous basal cell carcinoma, cutaneous squamous cell carcinoma and cutaneous melanoma is a polygenetic trait with each low-penetrance genetic variant contributing to the overall carcinogenic effect, and with extrinsic factors having the capacity to modify cancer risk by influencing the penetrance of the genetic variants.

### Ethical approval

Not required
